# Are autistic traits associated with suicidality? A test of the interpersonal‐psychological theory of suicide in a non‐clinical young adult sample

**DOI:** 10.1002/aur.1828

**Published:** 2017-07-07

**Authors:** M. K. Pelton, S. A. Cassidy

**Affiliations:** ^1^ Centre for Research in Psychology Behaviour and Achievement, Coventry University Coventry UK; ^2^ School of Psychology University of Birmingham Birmingham UK

**Keywords:** autistic traits, broader autism phenotype, suicide, suicidality, interpersonal psychological theory of suicide, autism spectrum conditions

## Abstract

Autism spectrum conditions (ASC) has recently been associated with increased risk of suicidality. However, no studies have explored how autistic traits may interact with current models of suicidal behavior in a non‐clinical population. The current study therefore explored how self‐reported autistic traits interact with perceived burdensomeness and thwarted belongingness in predicting suicidal behavior, in the context of the Interpersonal‐Psychological Theory of Suicide (IPTS). 163 young adults (aged 18–30 years) completed an online survey including measures of thwarted belonging and perceived burdensomeness (Interpersonal Needs Questionnaire), self‐reported autistic traits (Autism Spectrum Quotient), current depression (Centre for Epidemiological Studies Depression Scale), and lifetime suicidality (Suicide Behavior Questionnaire‐Revised). Results showed that burdensomeness and thwarted belonging significantly mediated the relationship between autistic traits and suicidal behavior. Both depression and autistic traits significantly predicted thwarted belonging and perceived burdensomeness. Autistic traits did not significantly moderate the relationship between suicidal behavior and thwarted belonging or perceived burdensomeness. Results suggest that the IPTS provides a useful framework for understanding the influence of autistic traits on suicidal behavior. However, the psychometric properties of these measures need be explored in those with clinically confirmed diagnosis of ASC. ***Autism Res*** *2017, 10: 1891–1904*. © 2017 The Authors Autism Research published by International Society for Autism Research and Wiley Periodicals, Inc.

**Lay Summary:**

Recent research has shown that people with high autistic traits are more likely to attempt suicide. However, no studies have explored why. We found that people with high autistic traits were more likely to experience feelings that they do not belong in the world, are a burden on others, and depression, which may increase their likelihood of attempting suicide. These results suggest that promoting inclusion and independence in those with high autistic traits could help prevent people attempting suicide.

## Introduction

Suicide is the leading cause of death for men and women in the UK aged between 20 and 34 [Office for National Statistics, 2015]. Often termed a ‘preventable’ form of death, suicide is a complex phenomenon influenced by biological, social, and environmental factors making it stubbornly hard to predict [Walter & Pridmore, 2012; World Health Organization, 2012]. Recent research indicates an association between autistic traits and suicidality in clinical samples with and without diagnosis of an Autism Spectrum Condition (ASC) [Cassidy et al., 2014; Takara & Kondo, 2014]. However, no studies have yet explored associations between autistic traits and suicidality in a non‐clinical population, or how existing models of suicidality may interact with autistic traits (Cassidy and Rodgers, 2017). This is crucial to understand why people with high autistic traits may be at increased risk of suicide, and develop new effective assessment, support and treatment approaches for these individuals.

Autistic traits characteristic of ASC are normally distributed in the general population without an ASC diagnosis, termed the broader autism phenotype [Piven, Palmer, Jacobi, Childress, & Arndt, 1997]. For example, individual differences in ability to interact and communicate with others, imagination, attention to detail, and tendency to have narrow obsessive interests [Baron‐Cohen, Wheelwright, Skinner, Martin, & Clubley, 2001; Ruzich et al., 2015a]. The employment of a dimensional rather than categorical approach reflects current thinking that ASC diagnosis represents the extreme end of cognitive and behavioral differences distributed at various levels across the general population [Constantino & Todd, 2005; Hoekstra, Bartels, Hudziak, Van Beijsterveldt, & Boomsma, 2007; Baron‐Cohen et al., 2001; Sucksmith, Roth, & Hoekstra, 2011]. Such traits are recognized to increase susceptibility to poor social and functioning outcomes and reduced coping strategies at sub‐clinical as well as clinical levels [Best, Moffat, Power, Owens, & Johnstone, 2008; Kanne, Christ, & Reiersen, 2009]. For example, difficulties in social and communication skills could increase risk of experiencing social isolation [Orsmond, Shattuck, Cooper, Sterzing, & Anderson, 2013], which could in turn lead to secondary depression [Cassidy et al., 2014]. Depression and social isolation are significant risk factors for death by suicide in the general population [e.g., Kasper, Schindler, & Neumeister, 1996; Barraclough, Bunch, Nelson, & Sainsbury, 1974]. Are autistic traits therefore also associated with increased risk of suicidality in a non‐clinical sample?

Research has shown that autistic traits increase risk of suicidality in those with clinically confirmed diagnosis of ASC [Cassidy et al., 2014], and depressed patients without ASC [Takara & Kondo, 2014]. These results suggest that increased levels of autistic traits in clinical populations with and without clinically confirmed ASC increase risk of experiencing suicidality. Additional evidence for an association between autistic traits and suicidality is that those with confirmed diagnosis of ASC, at the extreme end of the autism phenotype, are at significantly increased of suicidality compared to other groups. Up to 66% of adults with ASC have contemplated suicide in their lifetime, significantly higher than the general population (17%) and patients with psychosis (59%) [Cassidy et al., 2014]. Suicide is a leading cause of premature mortality in those with ASC [Hirvikoski et al., 2015]. However, it is unknown whether autistic traits are also a risk factor for suicidality in non‐clinical populations without a diagnosis of ASC, or how models explaining suicidality in the general population may interact with autistic traits in a non‐clinical sample. Many psychiatrists are not trained in recognizing autistic traits, which can impede patients' access to appropriate support and treatment to prevent death by suicide [Raja, Azzoni, & Frustaci, 2011]. Elucidating risk markers for suicide in individuals with high autistic traits would therefore aid assessment and treatment of suicide risk in a large section of the population.

**Figure 1:**
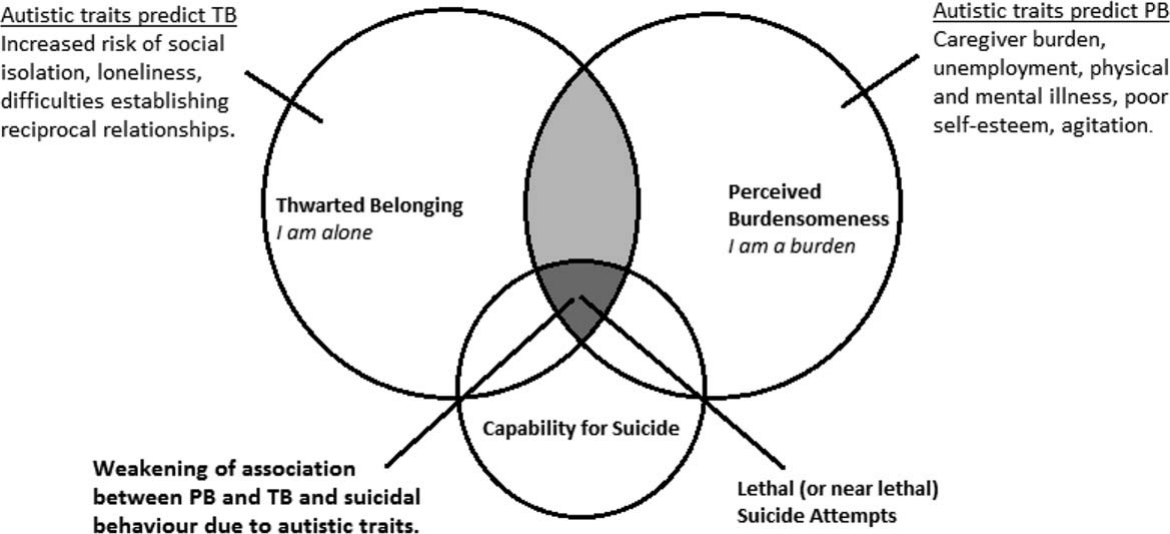
The interpersonal psychological theory of suicide and hypothesized interaction with autistic traits (adapted from Van Orden et al. 2010).

This study will be the first to explore these associations in relation to suicidal behavior in the broader autism phenotype. Due to its strong emphasis on social functioning, the framework of the Interpersonal‐Psychological Theory of Suicide (IPTS) is employed (Fig. 1). Non‐clinical samples are widely employed as proxy for clinical samples in model testing studies. Models to date have explored associations between autistic traits and (for example) social anxiety, loneliness, and social relationships [Jobe & White, 2007; Lamport & Zlomke, 2014], bullying [Kunihira, Senju, Dairoku, Wakabayashi, & Hasegawa, 2006] quality of life, stress levels and impaired coping styles [Pisula, Danielewicz, Kawa, & Pisula, 2015], and depressive symptoms [Jackson & Dritschel, 2016]. This study represents the first that has considered suicidal behavior, a high‐risk area for research where there are currently no models validated for use in ASC populations.

IPTS argues that the strongest predictors of suicidal behavior are mental health conditions, previous suicide attempts, social isolation, family conflict, unemployment, and physical illness. However, many experience these high‐risk factors and do not consider or attempt suicide [Van Orden et al., 2010]. The IPTS therefore argues that the social impact of negative life events and broader risk factors constitute distal risk factors for suicide. Suicide ideation is however activated via two proximal social risk factors: (i) an absence of reciprocal social relationships termed ‘thwarted belonging’; and (ii) the perception that one has become a hopeless burden on family and friends termed ‘perceived burdensomeness’ [Joiner, 2007; Van Orden et al., 2010]. These two factors constitute the focus of this study. The presence of a single social factor represents passive suicide ideation but the presence of both activates suicide ideation termed ‘the desire to die’. For a suicide attempt to be made a third variable ‘the acquired capability for suicide’ is required [Joiner, 2007; Van Orden et al., 2010]. See Figure 1.

The IPTS suggests that the nature of different mental health conditions influence their relationship with suicidal behavior [Joiner, 2007; Van Orden et al., 2010]. For example, conditions affecting social interaction and liability to others, such as depression, increase the likelihood that one will experience thwarted belonging and perceived burdensomeness [Davidson, Wingate, Grant, Judah, & Mills, 2011; Kleiman, Liu, & Riskind, 2014; Silva, Ribeiro, & Joiner, 2015]. Autistic traits have not been considered in IPTS research to date. However, observable behaviors which indicate the presence of the psychological state of thwarted belonging—self‐reported loneliness, few social supports, relationship breakdown, absence of life partner—are widely associated with high autistic traits [Causton‐Theoharis, Ashby, & Cosier, 2009; Chamberlain, Kasari, & Rotheram‐Fuller, 2007; Chen, Bundy, Cordier, Chien, & Einfeld, 2015; Chown & Beavan, 2012]. Additionally, observable behaviors indicative of perceived burdensomeness, such as unemployment [Riedel et al., 2015], physical illness [Hirvikoski et al., 2015], family burden [Cadman et al., 2012; Croen et al., 2015], low self‐esteem [Williamson, Craig, & Slinger, 2008], and agitation [e.g., McGonigle, Venkat, Beresford, Campbell, & Gabriels, 2014] are also associated with high autistic traits. Hence, it is possible that high levels of autistic traits may also be associated with increased suicidal behavior due to increased thwarted belonging and perceived burdensomeness.

There is also an established association between depression and suicidal behavior via thwarted belonging and perceived burdensomeness [Cole et al., 2013; Davidson et al., 2011; Joiner, 2007; Van Orden et al., 2010; Van Orden, Witte, Gordon, Bender, & Joiner, 2008]. Depression increases the experience of thwarted belonging due to symptoms of social disconnectedness. Individuals may experience difficulties with life achievements due to experiential avoidance, fatigue, motivational or concentration difficulties leading to increased feelings of burdensomeness [Davidson et al., 2011]. These may in turn increase perception of neediness leading to (inaccurate) negative projections of self onto those close by; a perception of being a hopeless burden to family and friends [Joiner, 2007].

The IPTS argues that co‐morbid diagnoses change the relationship with suicidal behavior due to an interaction of symptoms. Understanding these associations is important for designing effective clinical interventions [Silva et al., 2015]. For example, a diagnosis of co‐morbid depression and generalized anxiety disorder would increase suicide ideation but lower levels of capability reducing imminent risk [Silva et al., 2015]. Risk of depression increases with higher levels of autistic traits in the general population [Jackson & Dritschel, 2016]. Depression may also be exacerbated by the presence of autistic traits. For example, increased tendency for repetitive behavior, cognitive rigidity and difficulties in attention switching may increase risk of rumination (passive and internal problem solving without reference to solutions) [Cassidy et al., 2014; Gotham, Bishop, Brunwasser, & Lord, 2014]. Depressive symptoms and autistic traits may therefore interact; i.e., increased depressive symptoms may lead to observable increases in autistic traits [Andersen, Skogli, Hovik, Egeland, & Øie, 2015]. IPTS argues that the most dangerous form of thwarted belonging is where a limited social network and cognitive barriers to participation are accompanied by low mood [Van Orden et al., 2010]. Hence, increased levels of autistic traits may amplify the experience of depression leading to increased thwarted belonging. Therefore, one might expect that depression in the presence of high autistic traits together may more strongly predict higher levels of thwarted belonging than either of these two variables alone. By contrast, perceived burdensomeness relies on ability to put oneself in another's shoes, and adopt their perspective [termed a ‘theory of mind’; Baron & Frith, 1989] in order to successfully recognize that one constitutes a burden on others. Suicidal behavior in those with high autistic traits may therefore be hastened by the opposite process, that is, an inability to see that one's death will affect others [Hannon & Taylor, 2013]. Therefore autistic traits and depression may influence perceived burdensomeness independently of one another.

However, autistic traits are associated with difficulties that may impede ability to experience, and/or effectively report on feelings of thwarted belonging and perceived burdensomeness. For example, in order to believe that one is burden on others, you must first be able to take their perspective (they think I am not contributing), and attribute a belief (they think I am a burden on them) on the basis of this. Difficulties in taking another's perspective and subsequently attributing a belief, (termed a theory of mind) are commonly associated with high autistic traits and ASC [Baron‐Cohen, Leslie & Frith, 1989]. Hence it is possible that those with high autistic traits may have difficulty contemplating whether or not they are a burden on others. In the IPTS, thwarted belonging stresses social connectedness. Those with high autistic traits may avoid social situations and the anxiety this causes. The extent this impacts mental health may be affected by the extent to which the individual craves social connections. If the constructs of the IPTS do not effectively apply to the case of high autistic traits, then we would expect that the strength of the association between these factors with suicidality could be attenuated at high levels of autistic traits. This could offer some insight into a potentially different route to depression and suicide in those with high autistic traits.

The present study aimed to test these predictions, thereby addressing a gap in the literature in our understanding of how autistic traits in a non‐clinical sample may be associated with suicidal behavior [Hannon & Taylor, 2013; Segers & Rawana, 2014]. Firstly, this study will examine the relationship between autistic traits and suicidal behavior through the proximal risk factors for suicidal behavior set out in the IPTS [Joiner, 2007; Van Orden et al., 2010]. Specifically, this study hypothesizes that the relationship between autistic traits and suicidality will be significantly mediated by thwarted belonging and perceived burdensomeness. Secondly, this study will consider the well‐established relationships between depression and both thwarted belonging and perceived burdensomeness [e.g., Cole et al., 2013; Davidson et al., 2011], by examining whether these relationships are moderated by autistic traits. Specifically, this paper hypothesizes that depressive symptoms and autistic traits will interact to increase the experience of thwarted belonging. By contrast, perceived burdensomeness will be predicted by depressive symptoms and autistic traits but there will be no interaction. Lastly, this study will explore the well‐established respective relationships between thwarted belonging and perceived burdensomeness and suicidal behavior [Joiner, 2007; Van Orden et al., 2010], by considering whether these are moderated by high levels of autistic traits. Specifically, this paper hypothesizes that perceived burdensomeness and thwarted belonging will each constitute a weaker predictor of suicidal behavior at high levels of autistic traits.

## Method

### Participants

Participants were undergraduate students and members of the general population (*n* = 163, 65% female, aged 18–30 years (mean age = 21.58 years)) (Table 1).

**Table 1:** Participant Demographics

Gender	Number (%)
Male	55 (33.7)
Female	106 (65)
Prefer not to say	2 (1.2)
Accommodation	
Live in family home	53 (32.5)
Live alone	8 (4.9)
Live with housemates	81 (49.7)
Live with partner	18 (11)
Other	3 (1.8)
Work status	
Full time university	135 (82.8)
Full time employment	17 (10.4)
Part time employment	4 (2.5)
College full time	3 (1.8)
Not in education or employment	1 (0.6)
Other	3 (1.8)
Ethnicity	
White British	73 (44.8)
White Irish/other	22 (13.5)
Asian Indian/Pakistani	17 (10.4)
Other Asian	15 (9.2)
Black Caribbean/African/other	29 (17.8)
Mixed White and Asian/Black Caribbean	4 (2.4)
Other	3 (1.8)
Neurodevelopmental condition	
Autism	4 (2.5)
Dyspraxia	3 (1.8)
Epilepsy	3 (1.8)
Learning difficulty	5 (3.1)
Other neurodevelopmental condition	5 (3.1)
No neurodevelopmental condition	145 (89)
Mental health diagnosis	
Depression	33 (20.2)
Psychosis	1 (0.6)
Bipolar Disorder	1 (0.6)
No mental health diagnosis	117 (71.8)
Prefer not to say	10 (6.1)
Other (anxiety disorders)	7 (4.3)

Students were recruited via the Coventry University psychology research participation scheme and via advertising posters placed around campus. Members of the general population were recruited via the Cambridge Autism Research Database (CARD), posters placed in community organizations and via social media channels.

### Materials

***AQ***. The Autism‐Spectrum Quotient (AQ) is a 50‐item self‐report questionnaire [Baron‐Cohen et al., 2001]. Scores range from 0 to 50, with higher scores indicating higher levels of self‐reported autistic traits. The AQ has strong evidence for its psychometric properties, reliably distinguishing those with and without ASC [Baron‐Cohen et al., 2001; Ruzich et al., 2015a], and is an acceptable screening instrument prior to ASC diagnosis [Woodbury‐Smith, Robinson, Wheelwright, & Baron‐Cohen, 2005]. Alpha = .852.

***INQ‐15***. The Interpersonal Needs Questionnaire (INQ‐15) is a 15‐item self‐report questionnaire assessing the two interpersonal constructs that describe the mental state required for desire to die; ‘Thwarted Belonging’ and ‘Perceived Burdensomeness’ [Van Orden, Cukrowicz, Witte, & Joiner, 2012]. The INQ‐15 has strong evidence for its psychometric properties and has been validated in young adults [Van Orden et al., 2012]. For burdensomeness subscale alpha = .936. For belonging subscale alpha = .905.

***CESD‐R***. The Centre for Epidemiologic Studies Depression Scale Revised (CESD‐R) is a validated 20‐item scale used to assess severity of depressive symptoms in line with the American Psychiatric Association Diagnostic and Statistical Manual V [Eaton, Smith, Ybarra, Muntaner, & Tien, 2004 2004]. It comprises nine subscales: dysphoria (sadness), anhedonia (loss of interest), appetite, sleep, thinking/concentration, guilt, fatigue, agitation, and suicide ideation. Respondents indicate on a five‐point scale how many days in the last 2 weeks they have been experiencing each symptom [Eaton et al., 2004 2004]. Scores range from 0 to 60 with scores over 16 indicating clinically significant depression. The CESD‐R has strong evidence for its psychometric properties, and has been validated for use in general population samples [Van Dam & Earleywine, 2011]. Alpha = .949.

***SBQ‐R***. The Suicide Behaviors Questionnaire‐revised (SBQ‐R) is a validated 4‐item self‐report questionnaire that assesses lifetime suicidal behavior, suicide ideation over the past 12 months, threat of suicide attempt, and likelihood of suicidal behavior in the future. This study employed question 1 of the SBQ‐R: ‘Have you ever thought about or attempted to kill yourself?’ There are six possible responses from ‘never’ to ‘I have attempted to kill myself and really hoped to die’ with responses grouped into one of four categories: ‘non‐suicidal’, ‘suicidal ideation’, ‘suicide plan’, and ‘suicide attempt’. The first item of the SBQ‐R has been validated for use in general population samples to reliably distinguish those who have and have not previously attempted suicide [Osman et al., 2001].

***Demographic questions***. Participants indicated their age, ethnic origin, living and work status, developmental history, and mental health diagnoses (Table 1).

### Ethical Approval

The current study received ethical approval from Coventry University Psychology Ethics Committee, and was approved by the scientific advisory group at the Autism Research Centre, University of Cambridge, prior to recruiting participants registered in the Cambridge Autism Research Database (CARD).

### Procedure

Participants were invited to complete an online survey using Bristol Online Survey tool. Participants read the participant information and indicated informed consent to participate via an online form. Participants were fully briefed about the nature of the research, that they could skip questions that made them feel uncomfortable, and were provided information about relevant support services before and after taking part in the study.

## Results

### Analytic Approach

Anonymised data were exported into SPSS version 22 for analysis. Simple mediation models explored whether the relationship between autistic traits and suicidal behavior were mediated by perceived burdensomeness and thwarted belonging, using SPSS custom dialogue box PROCESS [Field, 2009; Hayes, 2013], as in previous similar studies [e.g., Cole et al., 2013]. Hierarchical regressions explored whether autistic traits moderated associations between depression with burdensomeness and belonging, and whether associations between thwarted belonging, perceived burdensomeness and lifetime suicidality were attenuated at high levels of autistic traits. The Johnson‐Neyman technique was employed to identify regions of significance in significant interaction effects.

### Descriptive Statistics

Data from the SBQ‐R was significantly skewed (skew = 3.38), which was not successfully corrected by either square root or log transformation. The INQ is designed to be non‐normally distributed in the general population as it measures experiences and feelings that are rare [Van Orden et al., 2012]. Analyses were therefore undertaken using bootstrapping techniques, a robust analysis technique which is reliable even when assumptions of a symmetric distribution are not met [Field, 2009]. Utilizing this robust analysis technique did not alter the pattern of results, with similar direction and magnitude of effects and statistical significance found using bootstrapping or normal analytic approach, therefore untransformed results are reported for ease of interpretation.

All variables—autistic traits, depression, belonging, and burdensomeness—were significantly correlated with lifetime suicidal behavior (Table 2). Mean AQ score in this sample was 18.2, similar to the general population mean of 16.7 [Ruzich et al., 2015b], and student sample mean of 17.6 [Baron‐Cohen et al., 2001]. Clinically relevant AQ scores (AQ ≥ 26) were reported by 15.7% of the sample. Prevalence of lifetime suicide plan was 19.6%, and suicide attempts 8.6% of the current sample, similar to prevalence rates found in general and university populations [2.5–10% suicide attempts; Nock et al., 2008; Kessler, Borges, & Walters, 1999; O'Carroll, 1992].

**Table 2:** Means, Standard Deviations and Inter‐Correlations for All Variables

Variable	AQ	Belonging	Burden	SBQ‐R	CESD‐R	Age	Gender
AQ	‐						
Belongingness	.460*	‐					
Burdensomeness	.288*	.657*	‐				
SBQ‐R	.280*	.474*	.613*	‐			
CESD‐R	.301*	.612*	.658*	.562*	‐		
Age	.128	−.007	−.216*	−.020	−.077	‐	
Gender	−.029	.046	.167	.205	.234	.027	‐
Mean	18.29	26.66	12.07	1.96	19.02	21.58	‐
Standard deviation	7.83	12.55	8.28	.97	17.08	3.04	‐
Range	40	54	36	3	75	12	‐

*Note. n* = 163. AQ = Autism Quotient; SBQ‐R = Suicidal Behaviors Questionnaire Revised (Question 1); CESD‐R = Centre for Epidemiological Studies Depression Rating Revised; Burden = Perceived Burdensomeness subscale of the Interpersonal Needs Questionnaire; Belonging = Thwarted Belonging subscale of the Interpersonal Needs Questionnaire.

*Significant correlations *P* < .05.

### Is the Relationship between Autistic Traits and Suicidal Behavior Mediated by Thwarted Belonging and Perceived Burdensomeness?

Mediation analysis was used to explore whether autistic traits were associated with suicidal behavior through burdensomeness and thwarted belonging, as it allows for skewed samples and non‐normal distribution through bootstrapping procedures [Field, 2009; Hayes, 2013]. Simple linear regressions showed that Autistic traits (*R* ^2^ = .078, F(1,161) = 13.68, *P* = .001), Perceived burdensomeness (*R* ^2^ = .376, F(1,161) = 96.921, *P* = .001), and Thwarted belonging (*R* ^2^ = .225, F(1,161) = 46.754, *P* = .001) significantly predicted lifetime suicidality.

**Figure 2:**
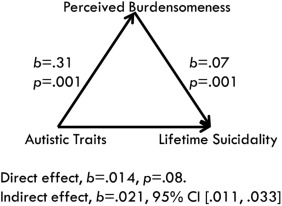
Model of the indirect effect of autistic traits on suicidal behavior through perceived burdensomeness.

**Figure 3:**
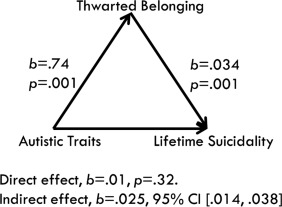
Model of the indirect effect of autistic traits on suicidal behavior through thwarted belonging.

Figures 2 and 3 below show the results of the path analysis. There was a significant indirect effect of autistic traits on suicidal behavior through perceived burdensomeness (*b* = .021 BCa CI [.011, .033]), with a medium effect (*k* ^2^ = .18, 95% BCa [.099, .269]). The direct effect of autistic traits on suicidal behavior became non‐significant once the mediator was added (*b* = .014, *P* = .08).

There was a significant indirect effect of autistic traits on suicidal behavior through thwarted belonging (*b* = .025, BCa CI [.014, .038]), with a medium effect (*k* ^2^ = .191 BCa CI [.106, .277]). The direct effect between autistic traits and suicidal behavior became non‐significant once the mediator was added (*b* = .01, *P* = .32).

### Do Autistic Traits Interact with Depressive Symptoms to Predict Thwarted Belonging and Perceived Burdensomeness?

Hierarchical multiple regressions were performed with thwarted belonging and perceived burdensomeness as the outcome variables. To statistically control for these variables, age and gender were entered into the first step. The second step comprised the main effects of autistic traits and depression, and the third step the interaction effect. All independent variables were mean centered to avoid issues of collinearity in the interaction effect [Field, 2009] (Table 3).

**Table 3:** Hierarchical Multiple Regression Predicting Thwarted Belonging from Depressive Symptoms and Autistic Traits

Independent variable	*F* for set	*R* ^2^	*t* for predictors	*df*	Semipartial correlation (sr)	*P*
1	.179	.002		2,160		.836
Age			−.109		−.009	.913
Gender			.591		.047	.555
2	34.008	.463		4,158		.001
Depression			8.443		.492	.001
Autistic traits			4.745		.277	.001
3	28.904	.479		5,157		.001
Autistic traits × depression			−2.241		−.129	.026

*Note*. Autistic traits = total AQ score. Depression = total CESD‐R score.

**Table 4:** Hierarchical Multiple Regression Equation Predicting Perceived Burdensomeness from Depressive Symptoms and Autistic Traits

Independent variable	*F* for set	*R* ^2^	*t* for predictors	*df*	Semipartial correlation (sr)	*P*
1	6.608	.076		2,160		.002
Age			−2.90		−.220	.004
Gender			2.272		.173	.024
2	35.920	.476		4,158		.001
Depression			9.427		.543	.001
Autistic traits			2.186		.126	.005
3	28.883	.479		5,157		.001
Autistic traits × depression			0.929		.054	.354

*Note*. Autistic traits = total AQ score. Depression = total CESD‐R score.

***Thwarted belonging***. In step 1, a model containing age and gender did not significantly predict thwarted belonging (F(2,160) = .179, *P* = .836). In step 2, depression and autistic traits each significantly predicted thwarted belonging (F(4,158) = 34.008, *P* = .001) accounting for 46.3% of the variance. In step 3, the addition of the interaction effect between autistic traits and depression significantly accounted for a further 1.6% of the variance (F(5, 157) = 28.904, *P* = .026).

**Figure 4:**
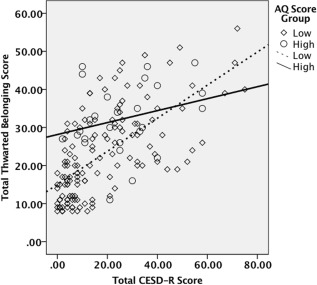
Interaction between depressive symptoms and autistic traits in the prediction of thwarted belonging.

This interaction effect is illustrated in the scatterplot in Figure 4. The relationship between depression and thwarted belonging is illustrated at high (≥26) and low (<26) levels of autistic traits. As depicted in Figure 4, the relationship between depression and thwarted belonging is stronger at low levels of autistic traits than at high levels of autistic traits.

***Perceived burdensomeness***. In step 1, a model containing age and gender significantly predicted perceived burdensomeness (F2,160) = 6.608, *P* = .002). In step 2, the main effect of depression and autistic traits significantly predicted perceived burdensomeness (F(2,158) = 35.920, *P* = .000) accounting for 47.6% of the variance. In step 3, the addition of the interaction effect between autistic traits and depression did not significantly increase the prediction of burdensomeness (F(1,141) = 28.883, *P* = .000).

### Are Associations between Perceived Burdensomeness and Thwarted Belonging with Suicidal Behavior Attenuated in Those with High Levels of Autistic Traits?

The PROCESS model for moderation was employed as it encompasses the Johnson–Neyman (JN) technique to identify regions of significance within the continuum of autistic traits, in this case to specifically assess whether there is any difference in effect at high (clinical) levels of autistic traits [Field, 2009; Hayes, 2013]. Simple slopes were analysed at high, mean, and low values of the moderator. The predictor variable (X) was either thwarted belonging or perceived burdensomeness, the moderator (M) in each case was autistic traits and the outcome variable was suicidal behavior (Y).

There was no significant moderating effect of autistic traits on the relationship between perceived burdensomeness and suicidal behavior (*b* = −.001, 95% CI [−.0024, .0024], *t* = −.705, *P* = .482). The output of the JN technique showed that in this sample perceived burdensomeness significantly predicted suicidal behavior at all levels of autistic traits.

The examination of simple slopes shows that burdensomeness significantly predicted suicidal behavior at high (1SD above mean), (*b* = .065, 95%CI [.047, 0.082], *t* = 7.21, *P* = .000), medium (*b* = .070, 95%CI [.055, .084], *t* = 9.28, *P* = .000) and low levels (1SD below mean) (*b* = .074, 95%CI [.052, .097], *t* = 6.58 *P* = .000) of autistic traits. The value of beta of the regression equation increased as autistic traits decreased but this did not reach statistical significance in the current sample.

There was no significant moderating effect of autistic traits on the relationship between thwarted belonging and suicidal behavior (*b* = −.0003, 95%CI [−.002, .002], *t* = −.269, *P* = .79). Examination of simple slopes shows that thwarted belonging significantly predicted suicidal behavior at high (1SD above mean), (*b* = .032, 95%CI [.007, .057], *t* = 2.51, *P* = .013, medium (*b* = .034, 95%CI [.020, .047], *t* = 4.84, *P* =.000), and low levels (1SD below mean) (*b* = .036, 95%CI [.021, .051], *t* = 4.72, *P* = .000) of autistic traits. Whilst the value of beta increased as autistic traits decreased this did not reach statistical significance in the current sample.

## Discussion

This study aimed to explore for the first time whether autistic traits are associated with suicidality in a non‐clinical young adult sample, and how autistic traits interact with two proximal factors of the IPTS in predicting suicidality; thwarted belongingness and perceived burdensomeness. Results showed that self‐reported autistic traits significantly predicted suicidal behavior. This extends the limited body of research showing associations between autistic traits and risk of suicidality in clinical samples with and without ASC [Takara & Kondo, 2014; Cassidy et al., 2014]. This finding is also consistent with research indicating that high levels of autistic traits could increase likelihood of experiencing risk factors for suicidality. For example, increased exposure to negative life events [Graetz, 2016; Matthews et al., 2015; Pisula et al., 2015; Schmidt et al., 2015], difficulties forming and maintaining social relationships and support networks [Causton‐Theoharis et al., 2009; Chamberlain et al., 2007; Chown & Beavan, 2012; Gray, 2012; Griffith, Totsika, Nash, & Hastings, 2012a]. Coping strategies to effectively deal with life challenges could also be reduced by difficulties in imagination, and ability to think flexibly [Pisula et al., 2015, Pollmann, Finkenauer, & Begeer, 2010; Schmidt et al., 2015].

In line with predictions, results showed that the relationship between autistic traits and suicidal behavior was mediated by the two social risk factors of the IPTS: perceived burdensomeness and thwarted belonging. These findings are consistent with the principles of the IPTS, which argue that distal risk factors, in this case autistic traits, influence suicidal behavior via the proximal risk factors: thwarted belonging and perceived burdensomeness [Joiner, 2007; Van Orden et al., 2010]. Models containing each factor recorded medium effect sizes indicating a promising insight into understanding how autistic traits may be associated with suicidal behavior. Results are thus consistent with the interpretation that autistic traits increase vulnerability to experiencing perceived burdensomeness and thwarted belonging.

Results showed a significant indirect effect of autistic traits on suicidal behavior through thwarted belonging in line with our predictions from the IPTS. Thwarted belonging places the absence of reciprocal social relations in a central role in suicidal behavior [Joiner, 2007; Van Orden et al., 2010]. This is consistent with the widely documented association between autistic traits and reduced reciprocity leading to self‐report loneliness and limited social network [Chamberlain et al., 2007; Gray, 2012; Griffith et al., 2012a; Usher, Burrows, Schwartz, & Henderson, 2015; van Ommeren, Begeer, Scheeren, & Koot, 2012; White & Roberson‐Nay, 2009]. Personal and social functioning challenges are attributed to the core difficulties of social cognition, which provide the stable barriers to social participation described in the IPTS [Joiner Jr et al., 2009; Joiner, 2007; Van Orden et al., 2010].

There was a significant indirect effect of autistic traits on suicidal behavior through perceived burdensomeness. This is in line with findings that autistic traits are associated with caregiver burden, unemployment and poor self‐esteem, core components of Van Orden et al's (2010) observable behaviors of perceived burdensomeness [Cadman et al., 2012; McGonigle et al., 2014; Scott, Falkmer, Girdler, & Falkmer, 2015; Yang & Wang, 2014]. The association of burdensomeness with suicidal behavior is highly pertinent due to its established relationship with lethal suicide methods [Ciubara et al., 2015; Hannon & Taylor, 2013; Segers & Rawana, 2014]. However, consideration should be given to the possibility that autistic traits may lead to under‐reporting of burdensomeness given known difficulties in understanding others’ point of view [termed a Theory of Mind; Baron‐Cohen, Leslie, & Frith, 1989]. This model could be expanded, in particular, to include aspects of other co morbid diagnoses, especially anxiety disorders, and environmental factors.

In line with predictions results showed that there was a significant interaction between depressive symptoms and autistic traits in the prediction of thwarted belonging. Both depression and high autistic traits are typified by social difficulties: social withdrawal, loneliness, isolation, and relationship difficulties. Autistic traits and depressive symptoms are also seen to exacerbate one another [Ghaziuddin, Ghaziuddin, & Greden, 2002; Gotham, Unruh, & Lord, 2015; Matson & Williams, 2014]. Those who find social interaction challenging are likely to avoid social interaction, struggle to express themselves and to seek support leading to increased depressive symptoms [Hallett, Ronald, Rijsdijk, & Happé, 2010]. However, the direction of interaction was surprising; increasing levels of autistic traits appeared to reduce rather than exacerbate the experience of thwarted belonging. This could indicate a diametric effect; that is, autistic traits alleviate symptoms of depression reversing negative social consequences, such as withdrawal. An alternative explanation would be that autistic traits may lead to under‐reporting on the thwarted belonging subscale of the Interpersonal Needs Questionnaire. Items such as ‘these days I belong’ or ‘these days, I'm close to other people’ require abstract language and pragmatics, which are core language difficulties associated with autistic traits [Roth, 2010]. This reflects current research reporting substantial challenges in measuring depression in the context of high autistic traits due to over‐lapping symptoms and increased rates of alexithymia [Bird & Cook, 2013; Cassidy et al., 2014; Gotham et al., 2015]. Hence, high levels of autistic traits could reduce the ability to effectively articulate one's feelings of thwarted belonging.

Autistic traits and depressive symptoms both significantly predicted perceived burdensomeness but there was no interaction effect between these two variables. This suggests that both depression and autistic traits provide separate, additive contributions to the experience of perceived burdensomeness. For example, autistic traits are particularly associated with increased support needs regardless of depressive symptoms, such as unemployment, poor self‐esteem, and caregiver burden [Hendricks, 2010; McDonough & Revell, 2010; Scott et al., 2015, Myers, Ladner, & Koger, 2011; Parsi & Elster, 2015; Arán‐Filippetti & Krumm, 2013; Bekhet, Johnson, & Zauszniewski, 2012; Cadman et al., 2012; Griffith, Totsika, Nash, Jones, & Hastings, 2012b]. By contrast deep depression is particularly associated with negative self‐perceptions, where feelings of poor self‐worth trigger excessive reassurance seeking that one is truly valued and subsequent rejection [Cole et al., 2013; Hill & Pettit, 2014, Jahn, Cukrowicz, Mitchell, Poindexter, & Guidry, 2015; Nsamenang, Webb, Cukrowicz, & Hirsch, 2013]. It is unlikely that autistic traits would exacerbate these types of interpersonal symptoms of depression that rely on reciprocity due to theory of mind difficulties. However, the concept of perceived burdensomeness is described as a form of ‘extreme social ineffectiveness’ leading to two behavioral components: firstly, the perception of being a burden on others, as described above and secondly, intense self‐hatred, poor self‐esteem, and agitation [Joiner, 2007; Van Orden et al., 2010]. In the presence of theory of mind difficulties it is conceivable that this second component may be present in the absence of verbal expression of the former. This would be consistent with internalizing symptoms, physiological, and behavioral changes noted to associate depressive symptoms with autistic traits [Ghaziuddin et al., 2002; Gotham et al., 2015; Matson & Williams, 2014]. Future research could consider to what extent this is the case and how effectively this second component is captured by current measures. This reflects broader calls in the literature to improve the reliability and validity of the measurement of burdensomeness through development of measures that do not rely solely on verbal self‐report [e.g., Hill & Pettit, 2014].

The current study predicted that high levels of autistic traits would attenuate the relationship between perceived burdensomeness and thwarted belonging with suicidality. However, no significant moderation effect was found. This suggests that the capacity of the IPTS in predicting suicidality remains consistent at both high and low levels of autistic traits in a non‐clinical sample. This echoes findings from a wide range of other clinical populations, which have found that the ‘desire to die’, represented by perceived burdensomeness and thwarted belonging, significantly predicted suicide ideation when controlling for other risk factors [Barzilay et al., 2015; Bryan, Hernandez, Allison, & Clemans, 2013]. The results showed a trend for decreasing strength in the relationship between burdensomeness/thwarted belonging and suicidal behavior at the highest level of autistic traits in the current sample. This reflects research indicating difficulties reporting emotional states in those with high autistic traits [Bird & Cook, 2013; Greenberg et al., 2006]. Difficulties in reporting emotional states, theory of mind difficulties and abstract language ability may lead to a different interpretation of statements such as ‘These days, the people in my life would be better off if I were gone’ or ‘These days I think I am a burden on society’.

This study has a number of strengths. It is the only study to date which has explored how autistic traits interact with well validated risk factors in predicting suicidal behavior, in a non‐clinical sample. The limited number of available studies in this area have all been small scale and/or utilized clinical samples, without systematically exploring risk or protective factors according to a well‐defined and validated theoretical framework [Cassidy and Rodgers, 2017; Segers & Rawana, 2014]. It therefore addresses an under‐researched area—suicidality and autistic traits, and contributes to the development of a robust theoretical framework to understand and explore suicide in those with and without ASC. This is crucial to explore whether autistic traits are a risk marker for suicide in the wider population, and why, prior to further application in those with confirmed diagnosis of ASC.

This study also has some limitations. It utilized measures of self‐reported autistic traits (AQ), suicidality (SBQ‐R), depression (CESD‐R), perceived burdensomeness and thwarted belonging (INQ). However, all have undergone substantial reliability tests and have excellent psychometric properties [Ruzich et al., 2015a, 2015b; Osman et al., 2001; Eaton et al., 2004 2004; Van Orden et al., 2012]. The study drew on a largely female undergraduate sample. This limits the generalizability of results to the wider general population, and possibly to those with ASC. However, recent research has indicated that ASC may be under‐diagnosed in females [Rynkiewicz et al., 2016; Rutherford et al., 2016], and that females diagnosed with ASC, without co‐morbid intellectual disability, are at higher risk of dying by suicide than males [Hirvikoski et al., 2015]. Hence, it is important to explore associations between autistic traits and suicidality in females, without intellectual disability as this is particularly high‐risk group for death by suicide in the ASC population. This study was cross‐sectional, hence results from the mediation analyses must be interpreted with caution. Future research will need to conduct longitudinal studies to confirm results from the mediation analyses in the current study, regarding whether perceived burdensomeness and thwarted belongingness mediate the association between autistic traits and suicidal behaviors. However, results from regression analyzes demonstrate that autistic traits, thwarted belonging and perceived burdensomeness are associated with each other, and all significantly predict suicidal behavior.

In summary, this is the first study to use well‐validated measures to explore the relationship between autistic traits and suicidal behavior within a robust theoretical framework: the IPTS. This study provides the first evidence of an association between autistic traits and suicidal behavior in a non‐clinical sample, and provides new avenues for exploring and understanding suicide in those with and without ASC. Within this study, autistic traits do not appear to influence the relationship between perceived burdensomeness and thwarted belonging with suicidality, suggesting that these constitute, on the face of it, accurate predictors of suicidal behavior. Individuals with high levels of autistic traits are more likely to experience depression, thwarted belonging, and perceived burdensomeness, thereby increasing their risk of attempting suicide. Clinicians need to be aware of autistic traits, how they manifest and lead to increased risk of suicide. Improving inclusion, sense of belonging, and independence in people with high levels of autistic traits, could reduce premature death by suicide in a significant number of people.

## Conflict of interest

The authors state that they have no conflict of interest to declare.
